# Modeling the accuracy of a novel PCR and antibody ELISA for African swine fever virus detection using Bayesian latent class analysis

**DOI:** 10.3389/fvets.2023.1079918

**Published:** 2023-02-23

**Authors:** Rachel Schambow, Luis G. Giménez-Lirola, Vu Duc Hanh, Lai Thi Lan Huong, Nguyen Thi Lan, Pham Hong Trang, Do Duc Luc, Ha Xuan Bo, Vo Dinh Chuong, Rolf Rauh, William Nelson, Juan Carlos Mora-Díaz, Albert Rovira, Marie R. Culhane, Andres M. Perez

**Affiliations:** ^1^Center for Animal Health and Food Safety, University of Minnesota, St. Paul, MN, United States; ^2^Department of Veterinary Population Medicine, College of Veterinary Medicine, University of Minnesota, St. Paul, MN, United States; ^3^Innoceleris LLC., Ames, IA, United States; ^4^Faculty of Veterinary Medicine, Vietnam National University of Agriculture, Hanoi, Vietnam; ^5^Faculty of Animal Science, Vietnam National University of Agriculture, Hanoi, Vietnam; ^6^Vietnam Department of Animal Health, Ministry of Agriculture and Rural Development, Hanoi, Vietnam; ^7^Tetracore, Inc., Rockville, MD, United States; ^8^Department of Veterinary Diagnostic and Production Animal Medicine, College of Veterinary Medicine, Iowa State University, Ames, IA, United States; ^9^Veterinary Diagnostic Laboratory, College of Veterinary Medicine, University of Minnesota, St. Paul, MN, United States; ^10^Secure Food Systems Team, University of Minnesota, St. Paul, MN, United States

**Keywords:** Bayesian latent class analysis, ELISA, PCR, African swine fever, diagnostic test, evaluation, Vietnam

## Abstract

**Introduction:**

Diagnostic test evaluation for African swine fever (ASF) in field settings like Vietnam is critical to understanding test application in intended populations for surveillance and control strategies. Bayesian latent class analysis (BLCA) uses the results of multiple imperfect tests applied to an individual of unknown disease status to estimate the diagnostic sensitivity and specificity of each test, forgoing the need for a reference test.

**Methods:**

Here, we estimated and compared the diagnostic sensitivity and specificity of a novel indirect ELISA (iELISA) for ASF virus p30 antibody (Innoceleris LLC.) and the VetAlert™ ASF virus DNA Test Kit (qPCR, Tetracore Inc.) in field samples from Vietnam by assuming that disease status 1) is known and 2) is unknown using a BLCA model. In this cross-sectional study, 398 paired, individual swine serum/oral fluid (OF) samples were collected from 30 acutely ASF-affected farms, 37 chronically ASF-affected farms, and 20 ASF-unaffected farms in Vietnam. Samples were tested using both diagnostic assays. Diagnostic sensitivity was calculated assuming samples from ASF-affected farms were true positives and diagnostic sensitivity by assuming samples from unaffected farms were true negatives. ROC curves were plotted and AUC calculated for each test/sample combination. For comparison, a conditionally dependent, four test/sample combination, three population BLCA model was fit.

**Results:**

When considering all assumed ASF-affected samples, qPCR sensitivity was higher for serum (65.2%, 95% Confidence Interval [CI] 58.1–71.8) and OF (52%, 95%CI 44.8–59.2) compared to the iELISA (serum: 42.9%, 95%CI 35.9–50.1; OF: 33.3%, 95%CI 26.8–40.4). qPCR-serum had the highest AUC (0.895, 95%CI 0.863–0.928). BLCA estimates were nearly identical to those obtained when assuming disease status and were robust to changes in priors. qPCR sensitivity was considerably higher than ELISA in the acutely-affected population, while ELISA sensitivity was higher in the chronically-affected population. Specificity was nearly perfect for all test/sample types.

**Discussion:**

The effect of disease chronicity on sensitivity and specificity could not be well characterized here due to limited data, but future studies should aim to elucidate these trends to understand the best use of virus and antibody detection methods for ASF. Results presented here will help the design of surveillance and control strategies in Vietnam and other countries affected by ASF.

## 1. Introduction

African Swine Fever (ASF) is arguably one of the most significant animal disease threats currently facing global pork production. ASF is a notifiable disease of swine caused by the ASF virus (ASFV), a large (175–215 nm), icosahedral, enveloped, double-stranded DNA arbovirus (1). ASFV only infects members of the *Suidae* family including domestic pigs and wild boar, and it is not a threat to human health. ASF was first reported in Kenya in 1921 and now is globally widespread throughout Eastern Europe, Asia, and Africa (2, 3). Notably, it was detected in the Dominican Republic and Haiti in 2021 (4). No effective treatments exist for ASF, and even though promising vaccine candidates have been developed, their safety is still under evaluation, and it may be some time until they are regularly available for widespread use (5, 6). ASF disease control relies on preventing introduction with effective biosecurity, passive and active surveillance, and early detection of potential outbreaks followed by quarantine and eradication through mass depopulation to avoid disease spread (7).

Successful ASF surveillance and control strategies rely on timely and accurate ASF diagnosis to prevent disease spread and avoid false-positive ASF misdiagnosis that can lead to unnecessary culling of pigs and disruption to industry. Diagnostic assays based on virus and antibody detection are useful for surveillance, provided their intended use is appropriately defined and their diagnostic performance evaluated prior to deployment. Evaluation of the diagnostic sensitivity (DSe) and specificity (DSp) is part of the World Organization for Animal Health (WOAH)'s pathway for test validation, with various methodologies approved for analysis (8). In initial assessments of DSe and DSp, ideally samples from positive and negative reference populations that are representative of the intended target population should be used (8). Estimation of DSe and DSp would then involve the use of a gold standard reference test to which the new test is compared to. Although this method may be acceptable in circumstances where reference tests with high accuracy are available, the use of samples of known infection status is ideal for precise and unbiased evaluation of the diagnostic performance of a test. However, when animals or samples of unknown status are used or when no suitable reference test is available, considerable bias may be introduced. This is often the case in field studies, where it may be possible to determine the status of the herd or farm but impossible to ascertain the true disease status at animal level. However, field samples may best represent the intended use of these diagnostic tests and provide a more accurate evaluation of their performance, and they are important in monitoring assay performance after initial validation (8). To address these concerns, latent class models were developed to provide a flexible alternative for diagnostic test evaluation (9, 10).

Latent class models use multiple imperfect reference tests applied simultaneously to one or more populations to estimate the DSp and DSe of each test (9). These models allow for uncertainty about the samples' true status, making them an appropriate fit for analysis of field samples. They also can address the potential conditional dependence between tests that have similar biological basis, providing a more accurate estimate of DSe and DSp (11, 12). Bayesian latent class models (BLCA) use a Bayesian framework to formally incorporate prior knowledge to estimate the posterior probability of each tests' DSe and DSp. The prior represents how likely one believes the hypothesis to be true before data has been collected. In BLCA models of diagnostic test accuracy, priors can be provided for estimates of the DSe and DSp of each test and the disease prevalence in each sampled population (13). The use of these models is supported by the WOAH, and their implementation has become more common in veterinary medicine over the past 20 years (14–16).

For tests that provide a continuous outcome, results can be dichotomized into “positive” and “negative” categories (8). Additional categories, such as “intermediate” or “suspect”, are sometimes used as well. These categorical designations are made by specifying cut-off points. DSe and DSp of a diagnostic test can be increased or decreased by modifying its cut-off points, but their relationship is inversely related. Thus, test developers must choose a cut-off point that balances the desired DSe and DSp of the diagnostic test for its intended purpose. Receiver operating characteristic (ROC) curves can be a useful analysis to compare DSe and DSp over different cut-off points (17). Additionally, the area under the ROC curve (AUC) provides an estimate of the diagnostic test's global accuracy across all assay values and can be used to compare different assays.

Typically, ASF test assessment and validation have been performed on experimental samples. However, ongoing ASF outbreaks in Southeast Asia provide a unique opportunity for field testing of novel diagnostic tests. Particularly, Vietnam confirmed its first outbreak of ASF in February 2019 on a backyard pig farm in Hung Yen Province (18). Since then, ASF spread to all 63 provinces in Vietnam and resulted in an estimated nearly 6 million pigs lost in 2019 (19). Despite continuous efforts from private and public stakeholders to control the disease, since 2020 up to the present day, new ASF outbreaks continue to be reported (20, 21). The objective of the this study was to estimate and compare the diagnostic performance of a novel indirect enzyme-linked immunosorbent assay (iELISA) for ASF serum antibodies (iELISA), developed by Innoceleris Ames, IA, USA and produced and commercialized by Tetracore (Rockville, MD, USA), and the VetAlert™ ASFV DNA Test Kit (qPCR, Tetracore) in both serum and oral fluid (OF) samples collected on farms in Vietnam. Because no gold standard reference test was assumed and true disease status of sampled individuals was unknown, we also aimed to compare estimates from BLCA modeling to those produced when assuming disease status is known based on history, location, clinical signs, and duration of the ASF outbreak in Vietnam.

## 2. Materials and methods

### 2.1. Study design—Sampling, populations

The study was a prospective, cross-sectional field study to evaluate the performance of the two diagnostic assays, qPCR and iELISA, on serum and OF samples in Vietnam. Samples were collected from dates 2019 to 2021. Selection of farms was not researcher-driven, but part of ongoing ASFV regulatory activities by the Vietnamese veterinary services. Farms were from 17 provinces (Bac Giang, Bac Ninh, Dong Nai, Ha Nam, Ha Noi, Ha Tay, Hai Duong, Hoa Binh, Hung Yen, Nam Dinh, Nghe An, Phu Tho, Son La, Thai Binh, Thai Nguyen, Vinh Phuc, and Yen Bai). Sample collection was performed on farms throughout Vietnam using outbreaks detected/reported by the farm's veterinarian and farm owner. ASF-acutely affected, chronically affected, and unaffected herds were targeted. Acutely affected farms were defined as those with pigs with severe clinical symptoms of ASF, chronically affected farms as those with pigs which had developed mild clinical symptoms of ASF for a period of time (~6 weeks−2 months), and unaffected farms as those with no clinical or laboratory history of ASF at the farm level. On farm, pigs were selected by the farm's veterinarian, and on ASF-affected farms specifically, animals exhibiting clinical signs consistent with ASF were targeted for sampling. All pigs on farms were eligible for sampling. Paired individual serum and OF samples were collected from 100 pigs on 30 acutely ASF-affected farms, 98 pigs on 37 chronically affected farms, and 200 pigs on 20 non-affected farms, for a total of 398 paired samples from 87 farms. The number of samples taken per acute or chronic farm ranged from 1 to 10, while 10 samples were consistently collected on each unaffected farm. Farm information was recorded at the time of sampling including the farm's province, farm type, animals per barn and pen, brief history of ASF on the farm, overall health status of the pigs, and general vaccination status. Each sampled pig's age group category was also recorded.

### 2.2. Sample collection and processing, ELISA, and PCR protocols

#### 2.2.1. Sample collection and processing

All animal sampling and activities were performed in accordance with guidelines of the animal ethics committee of Vietnam National University of Agriculture. Blood was collected *via* right jugular vein venipuncture using a 20 gauge or smaller needle and syringe, with needle size adjusted per pig age. The 8–10 mL of blood collected was placed into a glass vial sans anticoagulant. The blood was allowed to clot at room temperature. Clotted blood was transferred to the laboratory under refrigerated conditions by placing the vials on ice gel packs in a transport container for serum extraction at the laboratory. Clotted blood was centrifuged for 10 min at 1,000 × *g* (Allegra™64R Centrifuge, Beckman Coulter), the sera were separated and aliquoted into 2 mL cryogenic vials, then stored at −80°C until further use.

Pig OF samples were collected using dry cotton swabs (prepared on 25 cm wooden stick). The cotton swab was used to rub inside the oral cavity, tonsils and pharynx of pigs 5–6 times so that the fluid was absorbed. Afterwards, the cotton swabs were transferred into a tube with 1.5 mL of phosphate-buffered saline (PBS—Tablets, USA) pH 7.4 and transported to the lab in an ice box. In the lab, the wooden stick was removed, and the cotton tip submerged in PBS was transferred into a zip bag. The fluid was collected by compressing the cotton swab in the bag. Aliquots of OF were placed into 2 mL cryogenic vials and stored at −80°C until tested.

#### 2.2.2. qPCR for ASFV DNA detection

Viral DNA extractions from serum and OF samples were performed using a MagMAX-96 viral kit (Thermo Fisher Scientific) with KingFisher Flex 96 Deep-Well Magnetic Particle Processor (Thermo Fisher Scientific). For the specific detection of ASFV DNA, the ASF 2.0 PCR dry assay (Tetracore, Inc., Rockville, MD, United States) was used. The assay, including ready-to-use reagents, was provided in a dried-down format, which can be stored and shipped at room temperature. The reagents were rehydrated by adding 20 μL of the rehydration buffer (TC-9094-064, Tetracore) to each ASFV reaction tube. The tube was then kept at room temperature for 5 min to allow for rehydration of the dry reagents. After this step, the tube was briefly vortexed (Cleaver, Scientific Ltd., Rugby, United Kingdom) for 10 s to fully dissolve the dry reagents. The rehydrated reagents were then transferred to the reaction tube and 5 μL of the extracted sample was then added to the reaction tube and loaded on the real-time PCR Instrument (CFX96™Real-Time System, Bio-Rad). Each sample was tested following these thermal cycling conditions: 95°C for 2 min, 45 cycles of, 95°C for 15 s, 60°C for 60 s (Collecting Optical data in channel FAM). Serum and OF samples with Ct values <38 were considered as positive and containing ASFV DNA. Samples with Ct values ≥ 38 were considered as negative and not containing ASFV DNA (manufacturer-specified).

#### 2.2.3. ASFV iELISA for antibody detection

In the present study, an ASFV VP30-based iELISA was used that was originally designed by Innoceleris LLC. and produced and commercialized by Tetracore Inc. Samples were tested according to manufacturer's instructions. With the exception of the wash solution (provided 20X), all iELISA reagents and controls were provided ready-to-use. In brief, 100 μL (reaction volume) of pre-diluted serum (1:100) or OF (1:2) samples were transferred to the pre-coated iELISA plate. After 45 min incubation (19–22°C), plates were washed 5 times using 300 μL of 1X wash solution, then 100 μL of enzyme conjugate was added to each well and the plates incubated (19–22°C) for 30 min. Then, after another washing step, the reaction was visualized by adding 100 μL of TMB substrate to each well and the plates incubated for 10 min at 19–22°C. Thereafter, 100 μL of stop solution was added to stop the reaction and the plates were read (450 nm) with a spectrophotometer (Epoch2, BioTek). The optical density (OD) response was expressed as sample to positive (S/P) ratios using the equation below.


$$\text{S}/\text{P}=\frac{\text{Sample OD }-\text{ Average Negative Control OD}}{\text{Average Positive Control OD}.\;-\text{ Average Negative Control OD}}$$

Serum samples with S/P <1 considered as negative and not having ASFV antibodies, while samples with S/P ≥ 1 were considered as positive and having ASFV-specific antibodies (manufacturer-specified). OF samples with S/P <0.5 considered as not having ASFV antibodies, while samples with S/P ≥ 0.5 were considered as having ASFV-specific antibodies.

### 2.3. Statistical models

Two methods were used to evaluate the DSe and DSp of each test and sample type: (1) calculation assuming disease status in reference populations; and (2) Bayesian latent class analysis assuming imperfect tests and unknown disease status. For the first approach, it was assumed that all samples from the ASF-acute and ASF-chronic populations were disease-positive and that all samples from the unaffected farms were disease-negative. The continuous test values for each subject were dichotomized into positive and negative categories using the cut-offs described in Sections 2.2.2 and 2.2.3. The tests' DSe using serum or OF were estimated using the ASF-affected populations, and DSp were estimated using the unaffected population, by placing the data in a 2 × 2 table of disease and test status. DSe was calculated as the proportion of disease-positive samples that tested positive, while DSp was calculated as the proportion of disease-negative samples that tested negative (Table 1). Ninety-five percent confidence intervals were calculated using the specified Clopper-Pearson exact method. The tests' DSe was calculated individually for the acute and chronic populations, and then for both populations combined.

**Table 1:** Number of correctly classified test results using iELISA serum and oral fluid (OF) antibody tests and qPCR serum and OF DNA tests under the assumption that all samples from acute and chronically affected farms are ASF-positive and all samples from unaffected farms are ASF-negative.

**Population**	**iELISA, serum Ab**	**qPCR, serum DNA**	**iELISA, oral fluid Ab**	**qPCR, oral fluid DNA**
**Number of test positive samples**			
Acute (*n* = 100)	16	74	11	69
Chronic (*n* = 98)	69	55	55	34
Acute and Chronic Combined ASF-affected (*n* = 198)	85	129	66	103
**Number of test negative samples**				
ASF-unaffected (*n* = 200)	200	200	198	200

#### 2.3.1. Bayesian latent class model

**Figure 1:**
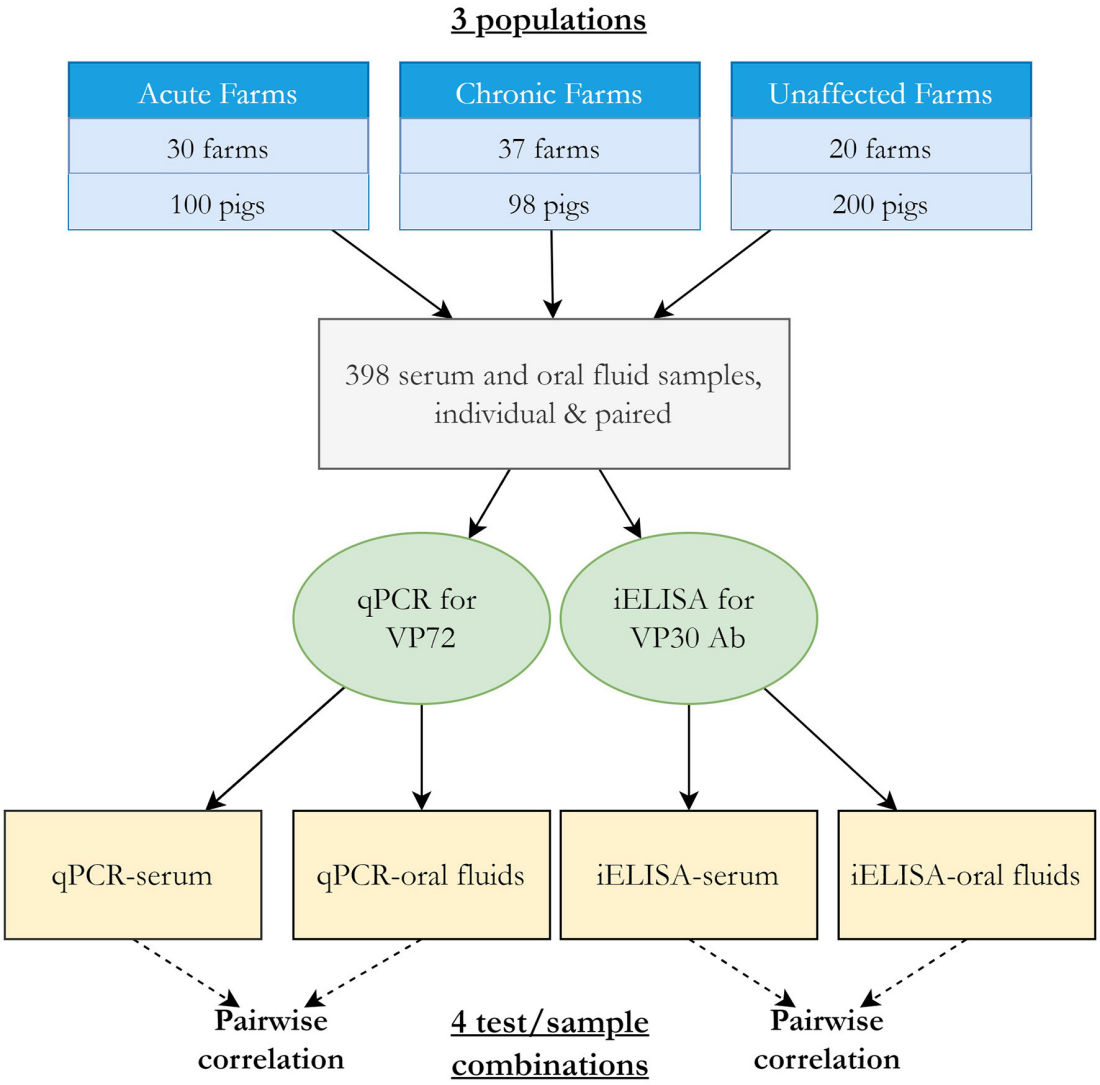
Overview of BLCA study design and model.

The Bayesian latent class analysis approach to evaluate DSe and DSp followed that of Dendukuri and Joseph (11) and Branscum et al. (13). A three population, four tests model with pairwise dependency was fit using the acute, chronic, and unaffected populations and each test-sample type combination (Figure 1). The full model is available in Supplementary material 1. Guidelines for reporting studies of diagnostic test accuracy using BLCMs were followed (16) (Supplementary material 2). Because both antibody and virus detection methods were modeled together, the latent class here represents infection where viral DNA is present yet is prolonged enough for antibodies to have been produced.

Covariance was modeled between samples tested by the same diagnostic test (i.e., between serum and OF samples tested by iELISA for ASFV antibodies and between serum and OF samples tested by qPCR for viral DNA, Figure 1), resulting in four pairwise comparisons. This accounts for the expectation that tests that use similar biological basis (e.g., detection of ASFV antibody) may be correlated, meaning the event that they classify individuals of infected or uninfected status the same will occur more than by chance. The covariance structure was parameterized using the approach described by (11) between the DSe or DSp for each pair of tests, where a Beta (1,1) prior was used to constrain the covariance estimates to positive values. The overall correlation of test outcome between pairs of tests for infected (rhoD) and uninfected (rhoDc) was calculated as described in Branscum et al. (13).

Prior values for each tests' DSe and DSp using serum and OF were estimated using previously published reports of the tests' performance, experimental studies, and through consultation with test designers and experts where data was limited (Table 2). In published reports, the VetAlert™ qPCR has consistently demonstrated high or perfect DSe and DSp across many different sample types (23, 24), however OF were not tested. Based on studies where other ASF DNA PCRs were evaluated using OFs, it was assumed that DSe of the VetAlert™ qPCR using OF would still be high but reduced compared to sample types such as whole blood (25, 26). The iELISA used here was recently evaluated in experimentally ASF-infected pigs in Russia using both serum and OF samples across 14 weeks (22). Raw data from those experiments were used directly to estimate priors by estimating an average DSe and DSp across timepoints and calculating 95% Clopper-Pearson exact confidence intervals. Additional results from samples collected through regulatory veterinary diagnostic laboratory testing in the United States, currently free of ASF, were also used to support the assumption of high DSp of the iELISA in serum and OF (Gimenez-Lirola, unpublished data). Because each population (acute, chronic, unaffected) was created by sampling a few pigs from many farms of a known ASF-status, it was assumed that the disease status of individuals within each sample population was likely very uniform; i.e., near certain high prevalence in the acute and chronic populations, and near certain freedom in the unaffected population. Due to this sampling strategy, reports of ASF-prevalence in herds in Vietnam were not chosen for informing the prevalence priors, and subjective estimates from the experts and authors were used instead (Table 2). No data was available to estimate correlation between tests, so vague Beta (1,1) priors were used for all covariance parameters. Using the most likely value for each estimate and its 95% confidence interval, BetaBuster (1.0) software was used to obtain the α and β parameters of the beta distributions for each prior (27). Some of the initial prior distributions for DSe and DSp were very narrow and resulted in non-convergence of the model, so the lower 95% confidence value was decreased by 10% for DSe and DSp estimates when obtaining the beta parameters in BetaBuster. A hyperprior of the form Bernoulli (0.001) was used to inform the prior value of the ASF-unaffected population.

**Table 2:** Values and sources of priors used for BLCA model.

**Test and sample type**	**DSe/DSp**	**Most likely**	**95% CI**	**α, β parameters**	**References**
iELISA, serum Ab	DSe	0.82	0.55, 0.99	9.20, 2.80	(22), Gimenez-Lirola, unpublished data
	DSp	0.99	0.89–1	30.29, 1.30	
qPCR, serum	DSe	1	0.88–1	23.43, 1	(23, 24)
	DSp	1	0.78–1	12.06, 1	
iELISA, oral fluid Ab	DSe	0.48	0.25–62.6	5.45, 5.82	(22), Gimenez-Lirola, unpublished data
	DSp	0.99	0.89-1	30.29, 1.30	
qPCR, oral fluids	DSe	0.95	0.5–1	4.77, 1.20	(23–25)
	DSp	1	0.79–1	12.71, 1	
ASF-acute population		1	0.1–1	1.30, 1	
ASF-chronic population		1	0.1–1	1.30, 1	
ASF-uninfected population		0.01	0.002–0.058	1.88, 88.28	
Covariance between iELISA serum and oral fluid, infected subjects				1, 1	
Covariance between qPCR serum and oral fluid, infected subjects				1, 1	
Covariance between iELISA serum and oral fluid, uninfected subjects				1, 1	
Covariance between qPCR serum and oral fluid, uninfected subjects				1, 1	

95% CI for diagnostic Se/Sp (DSe/DSp) estimates represents relaxed lower CI values.

The dichotomized test results were cross-classified in a contingency table (Table 3) representing all the possible combinations of test results from the four tests (2^4^ = 16 possible test combinations). The data was modeled multinomial with respect to *p* (the probability of an individual having a particular combination of test results, i.e., the probability of each cell in the contingency table) and *n* (the size of each population sampled). The probability of a given combination of test results *p* was modeled using the prevalence of each population and each tests' DSe and DSp and pairwise covariances. Five parallel DSe and DSp schemes (only serum, only OF, only PCR, only iELISA, or all four tests/samples) were also calculated within the model. Parallel testing here referred to the interpretation of test results whereby an animal is considered positive if any of the simultaneously applied tests are positive and can only be considered negative if all simultaneously applied tests are negative. Parallel DSe and DSp were calculated for pairwise parallel testing schemes and for all four test/sample combinations, adjusting for correlation for test pairs that were considered dependent (iELISA serum and iELISA OF; qPCR serum and qPCR OF) following that of Branscum et al. (13) and Bates et al. (28).

**Table 3:** Cross-classified results of iELISA serum (iELISA-S) and OF (iELISA-OF) antibody (Ab) tests and qPCR serum (qPCR-S) and oral fluids (qPCR-OF) tests used in BLCA representing all 16 possible test combinations, for acute, chronic, and unaffected populations.

**Population**	**iELISA-OF Ab**	**iELISA-S Ab**	**qPCR-OF** +		**qPCR-OF -**	
			**qPCR-S** +	**qPCR-S –**	**qPCR-S** +	**qPCR-S –**
ASF-affected, acute (*n* = 100)	+	+	4	0	4	0
		–	2	0	1	0
	–	+	4	1	3	0
		–	33	25	23	0
ASF-affected, chronic	+	+	9	8	21	17
(*n* = 98)		–	0	0	0	0
	–	+	5	2	6	1
		–	6	4	8	11
ASF-unaffected (*n* = 200)	+	+	0	0	0	0
		–	0	0	0	2
	–	+	0	0	0	0
		–	0	0	0	198

The Bayesian latent class analysis was performed using the freely available software WinBUGS v1.4.3 within R using the R2WinBUGS package (29, 30). WinBUGS (Bayesian inference Using Gibbs Sampling) is a program that allows for Bayesian inference using Markov chain Monte Carlo (MCMC) methods. The model was performed using three MCMC chains over 50,000 iterations with an initial burn-in of 5,000 iterations to obtain an effective sample size of at least 10,000 for the parameters. To eliminate potential autocorrelation, thinning was applied where 1 in every 10 consecutive samples were selected. Each parameter in each MCMC chain had a different starting input to ensure full exploration of the probability space. Convergence was visually confirmed by examining traceplots and obtaining a Gelmin-Rubin statistic for each parameter (31). Autocorrelation was assessed by examining autocorrelation plots. Summary statistics were generated for the parameters from the posterior density plots, where the median value represented the 50th percentile and the 95% credibility intervals represented the 2.5th and 97.5th percentile values.

#### 2.3.2. Sensitivity analysis

Other model structures and prior distributions were explored to assess the robustness of the BLCA model (Supplementary material 3). To assess the effect of the priors on the posterior distributions, two alternative sets of priors were used: vague Beta (1,1) distributions or changing by 25% of their original value. Alternative covariance structures were also explored. Pairwise dependence was modeled between iELISA and PCR samples of the same type, i.e., between PCR-serum/iELISA-serum and PCR-OF/iELISA-OF. Additionally, the original model was modified by parameterizing the covariance distributions as uniform using their natural minimum and maximum (11), allowing covariance take negative values. Due to constraints imposed on the MCMC sampler by the hyperprior for the unaffected population's prevalence, the effects of removing this hyperprior on convergence and parameter estimates were also explored.

An assumption of BLCA is equal performance of diagnostics tests across populations (9, 10). Because there was evidence that this assumption was potentially not met due to different performance in the acutely and chronically affected populations, a four test, two population model was fit where population one used the combined acute and chronic population data and population two used the unaffected population data. Two additional two-population models were fit where only the acute or chronic population data were used for population one, and the unaffected population data was used for population two. Results from these two models were compared to the DSe and DSp estimates when assuming infection status in acute and chronic populations only, respectively. For all the alternative analytical approximations, formulations, and parameterizations assessed in the sensitivity analysis here, variations of <10% in the point estimates and overlapping Bayesian credibility intervals (BCI) for the posterior values of the assessed parameters were considered evidence of robustness of the initial modeling approach.

### 2.4. ROC curves/AUC calculation

To understand how changes in cut-off values may affect DSe and DSp, ROC curves were produced and AUC calculated for each test-sample type combination using the package ROCR in R (17, 32). ASF-acute and -chronic population data were combined into one ASF-affected population, where all samples were assumed to be disease-positive. All ASF-unaffected farm samples were assumed to be disease-negative.

## 3. Results

### 3.1. Population and sample characteristics

Farms were distributed across 17 provinces mainly in Northcentral Vietnam and few in southern Vietnam. Most farms were located in Hung Yen (*n* = 22 farms) and Dong Nai (*n* = 8). In the acute population, 13 farms were classified as intensive and 17 as small holders, while in the chronic population, 14 farms were considered intensive and 22 as small holders. All ASF-unaffected farms were considered as industrial farms. As described in Vietnamese regulations, small holders contain from 10 to 29 animals, intensive farms contain from 30 to 299 animals, and industrial farms contain 300 or more animals (33). The age category of sampled pigs varied by population and farm. Of samples from acutely affected farms, 45 were grower pigs, 44 from sows, and 11 from weaned pigs. Samples from chronically affected farms were comprised of 28 growers, 6 finishers, 13 mature, 47 sows, and 4 weaned pigs. On ASF unaffected farms, 90 samples came from sows and 110 samples from weaned pigs. Sampled weaned pigs across farm types were of 3–5 weeks of age. Overall, no farms reported any type of ASF-vaccine usage. Other vaccine usage varied between farms, with many vaccinating for some combination of circovirus, parvovirus, foot-and-mouth disease (FMD) virus and/or classical swine fever (CSF) virus. Few farms also vaccinated for Porcine Reproductive and Respiratory Syndrome (PRRS).

### 3.2. Estimates under assumption of true disease status in populations

The qPCR had greatly increased DSe (serum: 74%, 95% CI 64.3–82.3; OF: 69%, 95% CI 58.97–77.9) compared to the iELISA (serum: 16%, 95% CI 9.43–24.7; OF: 11%, 95% CI 5.62–18.8) for both sample types in the acutely affected population and moderately increased DSe (serum: 65.2%, 95% CI 58.1–71.8; OF: 52%, 95% CI 44.8–59.2) compared to iELISA (serum: 42.9%, 95% CI 35.9–50.1; OF: 33.3%, 95% CI 26.8–40.4) in the combined populations (Table 4). The iELISA had higher DSe (serum: 70.4%, 95% CI 60.3–79.2; OF: 56.1%, 95% CI 45.7–66.1) compared to qPCR (serum: 56.1%, 95% CI 45.7–66.1; OF: 34.7%, 95% CI 25.4–44.98) with both sample types when considering only the chronically affected population. DSp was high for all test-sample combinations for all assays, with a minimal decrease in DSp for OF tested for antibodies by iELISA (99%, 95% CI 96.4–99.9).

**Table 4:** Estimates of diagnostic sensitivity (DSe) of ASFV iELISA and qPCR ASFV DNA assay for serum and oral fluid samples in acute, chronic, and combined-ASF-affected populations assuming true disease status, and diagnostic specificity (DSp) estimates in free-population, with 95% Clopper-Pearson confidence intervals.

**Test**	**Sample**	**Sensitivity, acutely affected**	**Sensitivity, chronically affected**	**Sensitivity, combined affected**	**Specificity, negative, i.e., unaffected**
iELISA for ASFV Ab	Serum	16 (9.43–24.7)	70.4 (60.3–79.2)	42.9 (35.9–50.1)	100 (98.2–100)
	Oral fluids	11 (5.62–18.8)	56.1 (45.7–66.1)	33.3 (26.8–40.4)	99 (96.4–99.9)
qPCR for ASFV DNA	Serum	74 (64.3–82.3)	56.1 (45.7–66.1)	65.2 (58.1–71.8)	100 (98.2–100)
	Oral fluids	69 (58.97–77.9)	34.7 (25.4–44.98)	52.0 (44.8–59.2)	100 (98.2–100)

### 3.3. BLCA model

The BLCA model converged well for all parameters with minimal autocorrelation. Posterior prevalence estimates were high for the acutely and chronically affected populations and near zero for the unaffected population (Table 5). The model provided higher posterior median estimates of DSe for all four test-sample types (Table 5; iELISA-serum: 46.2%, 95% BCI 39.4–52.9; iELISA-OF: 36.0%, 95% BCI 29.7–42.9; qPCR-serum: 70.0% 95% BCI 63.6–76.0; qPCR-OF: 53.9%, 95% BCI 46.7–61.0) compared to the values calculated from the combined, acutely, and chronically affected populations assuming positive disease status (Table 4), though the overall trends of test performance were identical. Posterior DSp estimates were nearly identical between the two evaluation methods, and overall, 95% BCI were of similar width to the estimated 95% confidence intervals (Tables 4, 5). Pairwise correlation for infected subjects (rhoD13) was high between the iELISA using serum and OF and low-moderate for uninfected subjects (rhoDc13) though the latter had a very wide 95% BCI. RhoD24 was low between qPCR serum and OF samples, while RhoDc24 was low-moderate and, similarly to the iELISA pairwise correlation, had a very wide 95% BCI. Of the five parallel testing schemes assessed, using all four test and sample combinations resulted in the highest parallel DSe with only a small decrease in DSp. The parallel DSe and DSp of using only qPCR samples or using only serum samples were similar with overlapping 95% BCI. The use of only iELISA samples had the lowest parallel DSe.

**Table 5:** BLCA posterior estimates of iELISA and qPCR diagnostic sensitivity and specificity (%) in serum and oral fluid samples with 95% Bayesian credibility intervals (BCI).

**Parameter**		**Posterior estimates and 95% BCI**	
**Test**	**Sample**	**Sensitivity**	**Specificity**
ELISA for ASFV Ab	Serum	46.2 (39.4–52.9)	99.6 (98.1–99.97)
	OF	36.0 (29.7–42.9)	98.5 (96.5–99.6)
	rhoD13, serum and OF	73.0 (63.9–81.2)	
	rhoDc13, serum and OF	24.3 (1.23–70.0)	
PCR for ASFV DNA	Serum	70.0 (63.6–76.0)	99.6 (98.0–99.99)
	OF	53.9 (46.7–61.0)	99.6 (98.1–99.99)
	rhoD24, serum and OF	2.14 (0.09–9.78)	
	rhoDc24, serum and OF	29.2 (1.18–84.56)	
Prevalence ASF-affected, acute		99.3 (96.2–99.9)	
Prevalence ASF-affected, chronic		95.1 (87.6–99.6)	
Prevalence ASF-unaffected		0 (0–0)	
**Parallel testing scheme**		**Parallel sensitivity**	**Parallel specificity**
All test/samples		92.7 (90.0–94.6)	97.3 (94.7–98.9)
iELISA-serum and iELISA-OF		48.2 (41.3–54.8)	98.2 (95.9–99.4)
PCR-serum and PCR-OF		85.6 (81.2–89.1)	99.2 (97.4–99.9)
iELISA-serum and PCR-serum		83.9 (79.8–87.7)	99.0 (97.0–99.8)
iELISA-OF and PCR-OF		70.1 (64.7–76.1)	98.0 (95.6–99.3)

RhoD and RhoDc are the overall correlation between tests within the infected and uninfected subjects, respectively.

### 3.4. Sensitivity analysis

The sensitivity analysis showed that parameter estimates were robust to changes in priors, hyperpriors, and model structure across the additional six different four test, three or two population models that were explored (Supplementary material 3). Removing the hyperprior allowed for better convergence of the parameter for the unaffected population prevalence, but with similar final results. The two population model where acute and chronic population data were combined also provided similar posterior medians and overlapping 95% BCI as the three population model (Supplementary material 3). The two population models where acute and chronic data were modeled separately showed small increased estimates for some DSe parameters compared to those when assuming disease status, but 95% confidence intervals and 95% BCI overlapped.

### 3.5. ROC curve analysis

**Figure 2:**
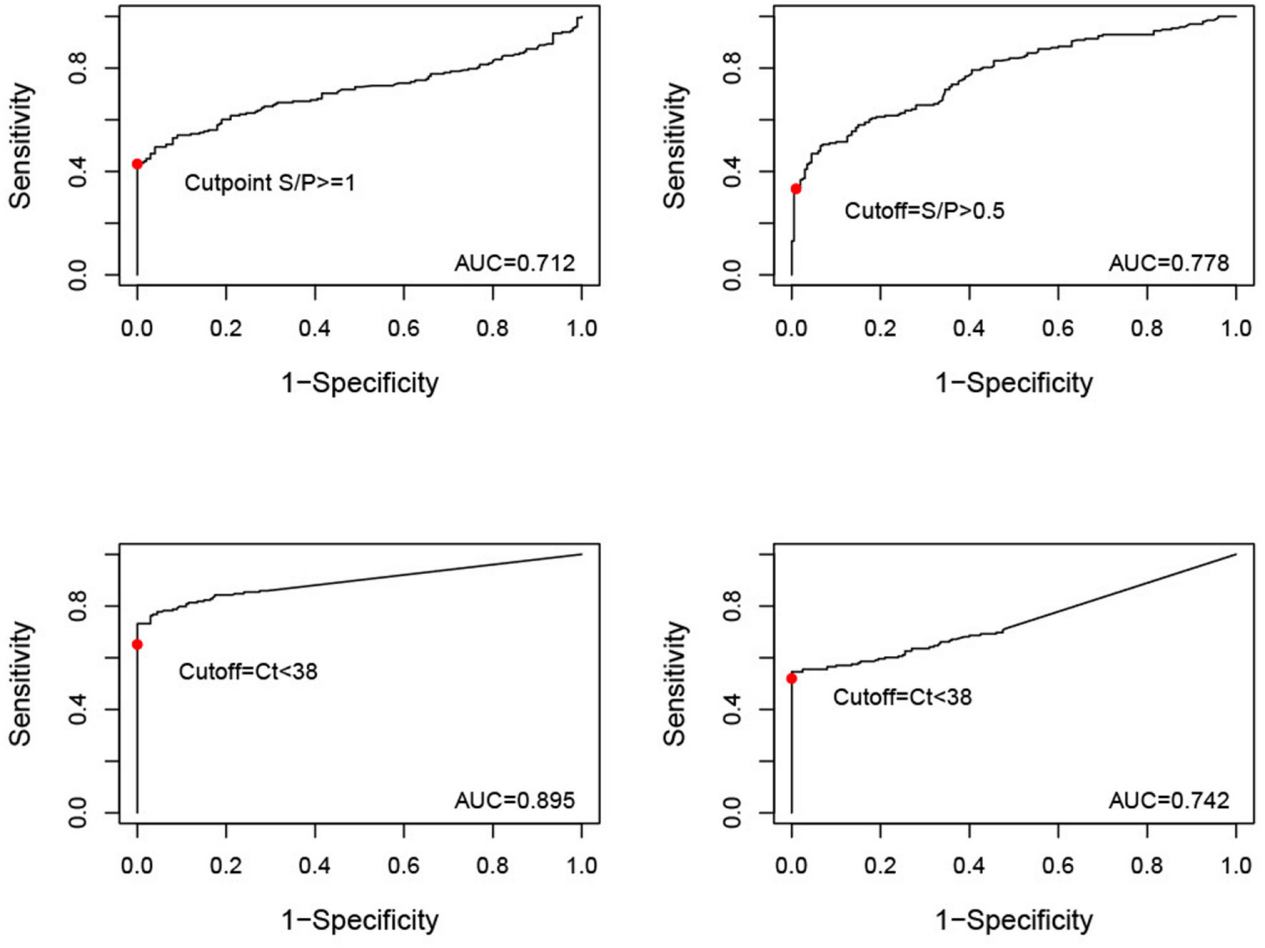
ROC curves (y = sensitivity, x = 1-specificity) for serum samples tested by iELISA, oral fluid (OF) samples tested by iELISA, serum samples tested by qPCR, and OF samples tested by qPCR. The solid red dot indicates the cut-off value used for analysis (iELISA serum positive = S/P ≥ 1, iELISA OF positive = S/P ≥ 0.5, qPCR serum and OF positive = Ct <38).

The best performing test according to its AUC value was the qPCR to detect ASF viral DNA in serum samples (Table 6). The remaining tests, i.e., qPCR to detect ASF viral DNA in OF and iELISA to detect ASFV antibodies in serum or OF, had similar AUC values with overlapping 95% confidence intervals. According to the ROC plots (Figure 2), decreasing iELISA cut-off or increasing PCR cut-off points to improve DSe would lead to substantial decreases in DSp with minimal gain in DSe.

**Table 6:** AUC and associated 95% confidence intervals for each test-sample type.

**Test**	**Sample**	**AUC (95% CI)**
ELISA for ASFV antibodies	Serum	0.712 (0.659–0.766)
	Oral fluids	0.778 (0.733–0.823)
PCR for ASFV DNA	Serum	0.895 (0.863–0.928)
	Oral fluids	0.742 (0.693–0.79)

## 4. Discussion

The ever-growing spread of ASF globally makes accurate and early ASFV detection critical to identify infected populations and successfully control disease outbreaks. Having reliable and accurate diagnostic assays not only improves detection but also reduces false positives, which is also an important component of an efficient disease response. ASF lacks any pathognomonic clinical signs and can present indistinguishably from diseases such as classical swine fever (CSF) or PRRS, among other systemic and hemorrhagic diseases, making confirmation of infection by diagnostic tests vitally important (34, 35). Here, we aimed to evaluate the DSe and DSp of two novel diagnostic tests, iELISA for ASFV antibody detection and qPCR for ASFV DNA detection, in both serum and OF samples from pigs under field conditions in Vietnam.

Overall, when population data was combined, qPCR had higher DSe than iELISA for both serum and OF samples. However, when considering acute or chronic populations separately, qPCR had higher DSe in the acute samples, while iELISA had higher DSe in chronic samples. The dynamics of ASFV viremia and the host's antibody response may explain in part the observed difference in DSe depending on infection stage. ASFV causes an initial viremia detectable by PCR within days after infection and is sustained for ~1 month. In pigs surviving the initial stages of infection, viremia decreases, and the amount of viral DNA present in the pig blood or sera may become too low to be reliably detected *via* viral DNA detection assays, but antibody testing allows for detection of the infection during and after cessation of viremia. ASFV antibodies become detectable ~7 days post-infection (dpi), per different antibody detection methods, including indirect immunoperoxidase, indirect immunofluorescence, and some ELISA (22, 36, 37). Furthermore, antibodies formed as a result of moderately virulent ASFV infections and/or in pigs that are in the chronic stage of ASFV infection are more reliably detected 13–14 days dpi. For example, in experimentally infected pigs, peak Cq values by qPCR were obtained by 18–19 dpi, with decreasing amounts of ASFV DNA in blood from 29 dpi onward until only 52% of pigs were qPCR-positive at 91 dpi (38). In contrast, in the same group of pigs, the percentage of ASFV-Ab positive animals and blocking values using the INGEZIM PPA COMPAC ELISA (Ingenasa) steadily increased from 10 dpi until reaching 99–100% by 63 dpi. In another study, pigs infected with ASFV through direct contact displayed a relatively steady decline in Ct values of a qPCR viral DNA tests of whole blood and oropharyngeal swabs from day 13 onward (39). In two surviving pigs, a strong antibody response was detected by blocking ELISA and IPT from 16 to 76 dpi. For PCR detection of ASF viral DNA, these dynamics may be further complicated by infections with moderate and low virulence ASFV strains, which may induce lower or inconsistent levels of viremia compared to highly virulent ASFV strains (39). These and the current results highlight the importance of using both virus and antibody detection methods in surveillance strategies to increase the probability of detection, as one method alone may fail to identify all infected pigs.

Another important characteristic of ASFV infection is its relatively slow within-herd spread. Though ASFV is sometimes considered to be highly contagious due to its high lethality, the initial number of infected pigs is low, and the transmission between infected and susceptible pigs is gradual compared to other high-consequence pathogens of swine like foot-and-mouth disease virus and CSF virus (40). For example, models of ASFV within-herd transmission have estimated delays in detection of weeks to months if only using mortality triggers, i.e., having a herd-level mortality that is higher than baseline, to initiate diagnostic investigation (41, 42). By this time, the infected population of pigs will be comprised of pigs at different time points of infection and various disease state durations. Thus, a limitation of the present study was the low number of pigs sampled per ASF-affected farms (<10), which definitively impacted the estimated DSe of the tests. The use of both virus and antibody detection diagnostic assay, along with an increased sample size will provide a higher probability of disease detection and confirmation. Again, this is of considerable importance in outbreaks caused by ASF strains of low or moderate virulence where pigs are more likely to survive the initial infection (39). Antibody-detection methods may be particularly useful and important in countries such as Vietnam, where partial culling methods are allowed (43). Using this approach, generally, farms are allowed to cull only ASF-affected animals and units rather than total depopulation. When unsuccessful, this technique can lead to farms being chronically affected with ASF, where antibody-detection would presumably be useful for herd-level surveillance.

The BLCA model's high correlation for test outcome in infected subjects between iELISA serum and OF antibodies is both expected and highlights the importance of incorporating test dependency in diagnostic test evaluation models. OF is made of components produced in buccal-associated tissues and from continuous exchange between the circulatory system and the buccal cavity through both passive and active processes (e.g., ultrafiltration, transudation, selective, and/or receptor mediated transport) (44). Consequently, what is detected in serum can often also be detected in OF. It is somewhat unexpected that the model estimated minimal correlation between PCR of detecting ASF viral DNA in serum and OF samples, and the reason behind this is unclear. This may be due to the method used to estimate the upper and lower boundaries for the correlation term, whereby high performing tests, such as the PCR-serum, result in low upper boundary values.

The similar AUC estimates and their overlapping CIs for samples tested by iELISA and OF tested by qPCR likely reflects their similar performance, depending on the tested population (e.g., acutely vs. chronically infected). AUC also indicated that serum samples tested by PCR performed the best overall. Long-lasting viremia may favor ASFV DNA detection across acute and chronic disease timepoints in serum compared to OF or antibody detection. Another consideration is that whole blood is the preferred diagnostic sample for PCR, having demonstrated a lower limit-of-detection compared to serum (45). This is likely because in blood ASFV is mostly associated with red blood cells (46). Detection *via* the PCR evaluated here may be improved when using whole blood samples. The ROC curves themselves indicate that the current cut-off values for test status established by the manufacturers are likely appropriate. For example, decreasing the cut-off value for iELISA in serum samples to 0.46 would achieve a modest increase in DSe to 56% in the current observed population, but at the cost of a decrease in DSp to 95%. It seems unlikely given the high consequences of a false positive for ASF that such a decrease in DSp would be acceptable for only minor improvements in Dse.

Based on our results and the particular experimental design of the present study, ELISA and qPCR diagnostic assays are best suited for herd-level surveillance. The high DSp of the tests presented here lends them to have few false positives, a critical need when performing surveillance in low-prevalence or disease-free regions to avoid extra unneeded, costly disease investigations. Field surveillance requires near-perfect DSp because any false positive results could cause significant disruptions to production (e.g., quarantines and no animal movements during the disease investigation) and financial consequences not only to the pork production system under investigation but to the whole swine industry if it resulted in a loss of ASF-free status, falling market prices, trade embargoes, or loss of consumer confidence and negative reactions to control measures (47, 48). This emphasizes the importance of establishing cut-off values that maximize DSp with minimal impact on DSe. Since DSe “evolves” dynamically during the course of infection (i.e., viremia decreases over time while antibodies increase over time) continuous active surveillance may reveal the true status at the next sampling time point.

When antibody- and virus-detection assays are combined in parallel testing schemes, these diagnostic assays may provide suitable herd-level DSe for surveillance strategies. Here, five parallel testing schemes were considered, varying from only serum, only OF, only PCR, only iELISA, or all four tests/samples being used. While the use of all four tests/samples resulted in an acceptable DSe (92.7%) for herd-level surveillance, it seems unlikely that this testing scheme would be realistic due to the costs of running multiple tests. It is likely more convenient to run both assays on the same sample type, as this would streamline sample collection, with the added benefit of assessing for presence of both virus and antibodies simultaneously. Using the values estimated in our BLCA model, parallel DSe using either serum (83.9%) or OF samples (70.1%) would likely be acceptable for herd-level testing with minimal loss in DSp. Another important consideration is that while in the present study OF were collected individually to correlate results with pig serum, typically OF samples are collected *via* pen-level sampling tools such as cotton ropes (49). This aggregate sample can be used to detect and monitor pathogens even at low prevalence, reducing both labor and cost associated with individual sampling. Future studies should consider evaluating aggregate OF samples as well to understand their potential for increasing the probability of ASF detection. Overall, animal health officials and producers would need to decide acceptable frequencies of false alarms and how to manage them before deploying any of these testing schemes. Future studies that incorporate test performance into disease transmission models could help to elucidate their potential use in surveillance and reduce uncertainty about their benefits and drawbacks.

The BCLA provided similar DSe and DSp estimates to those of the combined population data assuming true disease status. This may in part be due to the sampling methods used for selecting farms and individual animals. Only farms with unequivocal histories of ASF-status were chosen for sampling, and within ASFV-infected farms, animals with clinical signs were targeted. This created study populations for which we could be quite certain of the true disease status, although by all accounts the disease status was indeed assumed. BLCA models would likely more applicable for studies that can only be conducted on pigs of unclear/uncertain disease status or in herds with variable within-herd ASF prevalence.

Some assumptions of BLCA modeling may limit its use here. For example, BLCA assumes equal performance of tests across multiple populations (14). Based on our results, the tests had high variability of performance depending on the potential time point in disease. To understand how this and the prior choice may affect the model estimates, six other model structures and/or prior specifications were explored. Sensitivity analysis results were robust to these changes (Supplementary material 3), indicated by a <10% change in the posterior point estimates and overlapping BCI. Two additional two population, four test models were evaluated where the acute or chronic populations were included one at a time alongside the ASF-unaffected populations. These models were compared to the estimates from the ASF-acute or ASF-chronic specific DSe and DSp estimates when assuming disease status is known. Results showed minimal variability and overlapping BCI with the 95% confidence intervals (Table 4, ASF-acute and ASF-chronic). In future works, more complex analyses with detailed data that incorporate timepoint as a covariate into the model might help address this concern. Also, BLCA tends to be more difficult to apply to study designs using tests with different definitions of true disease status (e.g., presence of viral DNA vs. presence of antibodies) (14). Here, the tests' characterization of disease status varies considerably over time due to differing presence of viremia and antibodies. Despite these limitations, the ability to incorporate imperfect reference tests and correlation between tests likely has created less biased estimates of disease Se and Sp in the present study and is a strength of BLCA.

We acknowledge that there were some limitations in data collection that prevented further analyses. The sample collection did not follow a predefined scientific approach, but instead was a part of ongoing ASF regulatory activities. Consequently, the samples come with the limitations of field data, and ASF-status was assumed based on farm history. BLCA was used here to help obtain accurate DSe and DSp estimates despite these limitations. To obtain a suitable number of samples for analysis, many Vietnam farms were sampled (*n* = 87), but for many acutely and chronically affected farms, as few as 1–3 animals sampled per farm. This was most likely due to low within-herd prevalence of sick pigs to sample, the slow within-farm spread of ASFV, and the need to adequately represent differential stages of the infection across individual animals. The exact time point of infection for sampled pigs from acute and chronic farms was unknown, so it was difficult to assess the true impact of infection time point on test performance. It is unclear how much further in disease progression all chronic farms were compared to acute farms. As this is clearly an important factor to ASF test performance, future longitudinal studies with repeated sampling of individuals over time would be highly beneficial to understand these dynamics. Additionally, other important covariates of test status were not collected and could not be included in the analysis. Factors such as breed, presence of individual clinical signs, and accurate pig age all might be important for test performance but could not be considered in the present study. Finally, it is unknown how well these results may generalize to pigs of European or American origin; thus these tests may perform differently in other swine populations.

In summary, these results are an important evaluation of novel ASF diagnostic tests in field settings that provides real-world context about their performance. Animal health officials and epidemiologists can use these results to appropriately apply these diagnostic tests in surveillance and control strategies in Vietnam and other ASF-affected countries.

## Data availability statement

The original contributions presented in the study are included in the article/Supplementary material, further inquiries can be directed to the corresponding author.

## Ethics statement

Ethical review and approval was not required for the animal study because ASF continues to be a regulated and reportable disease in Vietnam. This study was IACUC exempted as it did not involve special sampling procedures that require ethical approval. Written informed consent for participation was not obtained from the owners because ASFV continues to be a regulated and reportable disease in Vietnam and sampling was conducted as a part of regular reporting and ASFV testing procedures.

## Author contributions

LG-L and AP designed the study. VC, NL, LH, VH, PT, DL, and HB coordinated sample collection, field testing, and laboratory analysis. LG-L assembled the data. LG-L, AP, MC, and AR helped in interpretation of the results. RS ran the statistical analyses and drafted the manuscript. All authors contributed to the article and approved the submitted version.
