# Supplementary material for: Modeling the accuracy of a novel PCR and antibody ELISA for African swine fever virus detection using Bayesian latent class analysis
Source: Front Vet Sci. 2023 Feb 23;10:1079918. doi: 10.3389/fvets.2023.1079918 (PMC9995851; doi:10.3389/fvets.2023.1079918)
Supplement: Supplementary file 1 [file Data_Sheet_1.docx]

model{

##############################

# likelihood of observed data

###############################

y1[1:Q, 1:Q, 1:Q, 1:Q] ~ dmulti(p1[1:Q, 1:Q, 1:Q, 1:Q], n1)

y2[1:Q, 1:Q, 1:Q, 1:Q] ~ dmulti(p2[1:Q, 1:Q, 1:Q, 1:Q], n2)

y3[1:Q, 1:Q, 1:Q, 1:Q] ~ dmulti(p3[1:Q, 1:Q, 1:Q, 1:Q], n3)

####################################################

# probabilities for four cross-classified tests (16)

#####################################################

p1[1,1,1,1] <- pr1*((s1*s3+covs13)*(s2*s4+covs24))+(1-pr1)*(((1-c1)*(1-c3)+covc13)*((1-c2)*(1-c4)+covc24))

p1[1,1,1,2] <- pr1*((s1*s3+covs13)*(s2*(1-s4)-covs24))+(1-pr1)*(((1-c1)*(1-c3)+covc13)*((1-c2)*c4-covc24))

p1[1,1,2,1] <- pr1*((s1*(1-s3)-covs13)*(s2*s4+covs24))+(1-pr1)*(((1-c1)*c3-covc13)*((1-c2)*(1-c4)+covc24))

p1[1,1,2,2] <- pr1*((s1*(1-s3)-covs13)*(s2*(1-s4)-covs24))+(1-pr1)*(((1-c1)*c3-covc13)*((1-c2)*c4-covc24))

p1[1,2,1,1] <- pr1*(s1*s3+covs13)*((1-s2)*s4-covs24) + (1-pr1)*(((1-c1)*(1-c3)+covc13)*(c2*(1-c4)-covc24))

p1[1,2,1,2] <- pr1*((s1*s3+covs13)*((1-s2)*(1-s4)+covs24))+(1-pr1)*(((1-c1)*(1-c3)+covc13)*(c2*c4+covc24))

p1[1,2,2,1] <- pr1*((s1*(1-s3)-covs13)*((1-s2)*s4-covs24))+(1-pr1)*(((1-c1)*c3-covc13)*(c2*(1-c4)-covc24))

p1[1,2,2,2] <- pr1*((s1*(1-s3)-covs13)*((1-s2)*(1-s4)+covs24))+(1-pr1)*(((1-c1)*c3-covc13)*(c2*c4+covc24))

p1[2,1,1,1] <- pr1*(((1-s1)*s3-covs13)*(s2*s4+covs24))+(1-pr1)*((c1*(1-c3)-covc13)*((1-c2)*(1-c4)+covc24))

p1[2,1,1,2] <- pr1*(((1-s1)*s3-covs13)*(s2*(1-s4)-covs24))+(1-pr1)*((c1*(1-c3)-covc13)*((1-c2)*c4-covc24))

p1[2,1,2,1] <- pr1*(((1-s1)*(1-s3)+covs13)*(s2*s4+covs24))+(1-pr1)*((c1*c3+covc13)*((1-c2)*(1-c4)+covc24))

p1[2,1,2,2] <- pr1*(((1-s1)*(1-s3)+covs13)*(s2*(1-s4)-covs24))+(1-pr1)*((c1*c3+covc13)*((1-c2)*c4-covc24))

p1[2,2,1,1] <- pr1*(((1-s1)*s3-covs13)*((1-s2)*s4-covs24))+(1-pr1)*((c1*(1-c3)-covc13)*(c2*(1-c4)-covc24))

p1[2,2,1,2] <- pr1*(((1-s1)*s3-covs13)*((1-s2)*(1-s4)+covs24))+(1-pr1)*((c1*(1-c3)-covc13)*(c2*c4+covc24))

p1[2,2,2,1] <- pr1*(((1-s1)*(1-s3)+covs13)*((1-s2)*s4-covs24))+(1-pr1)*((c1*c3+covc13)*(c2*(1-c4)-covc24))

p1[2,2,2,2] <- pr1*(((1-s1)*(1-s3)+covs13)*((1-s2)*(1-s4)+covs24))+(1-pr1)*((c1*c3+covc13)*(c2*c4+covc24))

p2[1,1,1,1] <- pr2*((s1*s3+covs13)*(s2*s4+covs24))+(1-pr2)*(((1-c1)*(1-c3)+covc13)*((1-c2)*(1-c4)+covc24))

p2[1,1,1,2] <- pr2*((s1*s3+covs13)*(s2*(1-s4)-covs24))+(1-pr2)*(((1-c1)*(1-c3)+covc13)*((1-c2)*c4-covc24))

p2[1,1,2,1] <- pr2*((s1*(1-s3)-covs13)*(s2*s4+covs24))+(1-pr2)*(((1-c1)*c3-covc13)*((1-c2)*(1-c4)+covc24))

p2[1,1,2,2] <- pr2*((s1*(1-s3)-covs13)*(s2*(1-s4)-covs24))+(1-pr2)*(((1-c1)*c3-covc13)*((1-c2)*c4-covc24))

p2[1,2,1,1] <- pr2*(s1*s3+covs13)*((1-s2)*s4-covs24) + (1-pr2)*(((1-c1)*(1-c3)+covc13)*(c2*(1-c4)-covc24))

p2[1,2,1,2] <- pr2*((s1*s3+covs13)*((1-s2)*(1-s4)+covs24))+(1-pr2)*(((1-c1)*(1-c3)+covc13)*(c2*c4+covc24))

p2[1,2,2,1] <- pr2*((s1*(1-s3)-covs13)*((1-s2)*s4-covs24))+(1-pr2)*(((1-c1)*c3-covc13)*(c2*(1-c4)-covc24))

p2[1,2,2,2] <- pr2*((s1*(1-s3)-covs13)*((1-s2)*(1-s4)+covs24))+(1-pr2)*(((1-c1)*c3-covc13)*(c2*c4+covc24))

p2[2,1,1,1] <- pr2*(((1-s1)*s3-covs13)*(s2*s4+covs24))+(1-pr2)*((c1*(1-c3)-covc13)*((1-c2)*(1-c4)+covc24))

p2[2,1,1,2] <- pr2*(((1-s1)*s3-covs13)*(s2*(1-s4)-covs24))+(1-pr2)*((c1*(1-c3)-covc13)*((1-c2)*c4-covc24))

p2[2,1,2,1] <- pr2*(((1-s1)*(1-s3)+covs13)*(s2*s4+covs24))+(1-pr2)*((c1*c3+covc13)*((1-c2)*(1-c4)+covc24))

p2[2,1,2,2] <- pr2*(((1-s1)*(1-s3)+covs13)*(s2*(1-s4)-covs24))+(1-pr2)*((c1*c3+covc13)*((1-c2)*c4-covc24))

p2[2,2,1,1] <- pr2*(((1-s1)*s3-covs13)*((1-s2)*s4-covs24))+(1-pr2)*((c1*(1-c3)-covc13)*(c2*(1-c4)-covc24))

p2[2,2,1,2] <- pr2*(((1-s1)*s3-covs13)*((1-s2)*(1-s4)+covs24))+(1-pr2)*((c1*(1-c3)-covc13)*(c2*c4+covc24))

p2[2,2,2,1] <- pr2*(((1-s1)*(1-s3)+covs13)*((1-s2)*s4-covs24))+(1-pr2)*((c1*c3+covc13)*(c2*(1-c4)-covc24))

p2[2,2,2,2] <- pr2*(((1-s1)*(1-s3)+covs13)*((1-s2)*(1-s4)+covs24))+(1-pr2)*((c1*c3+covc13)*(c2*c4+covc24))

p3[1,1,1,1] <- pr3*((s1*s3+covs13)*(s2*s4+covs24))+(1-pr3)*(((1-c1)*(1-c3)+covc13)*((1-c2)*(1-c4)+covc24))

p3[1,1,1,2] <- pr3*((s1*s3+covs13)*(s2*(1-s4)-covs24))+(1-pr3)*(((1-c1)*(1-c3)+covc13)*((1-c2)*c4-covc24))

p3[1,1,2,1] <- pr3*((s1*(1-s3)-covs13)*(s2*s4+covs24))+(1-pr3)*(((1-c1)*c3-covc13)*((1-c2)*(1-c4)+covc24))

p3[1,1,2,2] <- pr3*((s1*(1-s3)-covs13)*(s2*(1-s4)-covs24))+(1-pr3)*(((1-c1)*c3-covc13)*((1-c2)*c4-covc24))

p3[1,2,1,1] <- pr3*(s1*s3+covs13)*((1-s2)*s4-covs24) + (1-pr3)*(((1-c1)*(1-c3)+covc13)*(c2*(1-c4)-covc24))

p3[1,2,1,2] <- pr3*((s1*s3+covs13)*((1-s2)*(1-s4)+covs24))+(1-pr3)*(((1-c1)*(1-c3)+covc13)*(c2*c4+covc24))

p3[1,2,2,1] <- pr3*((s1*(1-s3)-covs13)*((1-s2)*s4-covs24))+(1-pr3)*(((1-c1)*c3-covc13)*(c2*(1-c4)-covc24))

p3[1,2,2,2] <- pr3*((s1*(1-s3)-covs13)*((1-s2)*(1-s4)+covs24))+(1-pr3)*(((1-c1)*c3-covc13)*(c2*c4+covc24))

p3[2,1,1,1] <- pr3*(((1-s1)*s3-covs13)*(s2*s4+covs24))+(1-pr3)*((c1*(1-c3)-covc13)*((1-c2)*(1-c4)+covc24))

p3[2,1,1,2] <- pr3*(((1-s1)*s3-covs13)*(s2*(1-s4)-covs24))+(1-pr3)*((c1*(1-c3)-covc13)*((1-c2)*c4-covc24))

p3[2,1,2,1] <- pr3*(((1-s1)*(1-s3)+covs13)*(s2*s4+covs24))+(1-pr3)*((c1*c3+covc13)*((1-c2)*(1-c4)+covc24))

p3[2,1,2,2] <- pr3*(((1-s1)*(1-s3)+covs13)*(s2*(1-s4)-covs24))+(1-pr3)*((c1*c3+covc13)*((1-c2)*c4-covc24))

p3[2,2,1,1] <- pr3*(((1-s1)*s3-covs13)*((1-s2)*s4-covs24))+(1-pr3)*((c1*(1-c3)-covc13)*(c2*(1-c4)-covc24))

p3[2,2,1,2] <- pr3*(((1-s1)*s3-covs13)*((1-s2)*(1-s4)+covs24))+(1-pr3)*((c1*(1-c3)-covc13)*(c2*c4+covc24))

p3[2,2,2,1] <- pr3*(((1-s1)*(1-s3)+covs13)*((1-s2)*s4-covs24))+(1-pr3)*((c1*c3+covc13)*(c2*(1-c4)-covc24))

p3[2,2,2,2] <- pr3*(((1-s1)*(1-s3)+covs13)*((1-s2)*(1-s4)+covs24))+(1-pr3)*((c1*c3+covc13)*(c2*c4+covc24))

####################################################################

# prior distributions of prevalence, sensitivities, and specificities

####################################################################

pr1~dbeta(alpha.pi1,beta.pi1) ## prevalence for population 1

pr2 <- Z*pi2star ## prevalence for population 2

pr3~dbeta(alpha.pi3,beta.pi3) ## prevalence for population 3

pi2star ~ dbeta(alpha.pistar2,beta.pistar2) ## hyperprior for population 2

Z ~ dbern(tau1)

tau1 <- 0.001

c1~dbeta(alpha.spec1, beta.spec1) ## Specificity ELISA serum

c2~dbeta(alpha.spec2, beta.spec2) ## Specificity PCR serum

c3~dbeta(alpha.spec3, beta.spec3) ## Specificity ELISA oral fluids

c4~dbeta(alpha.spec4, beta.spec4) ## Specificity PCR oral fluids

s1~dbeta(alpha.sens1,beta.sens1) ## Sensitivity ELISA serum

s2~dbeta(alpha.sens2,beta.sens2) ## Sensitivity PCR serum

s3~dbeta(alpha.sens3,beta.sens3) ## Sensitivity ELISA oral fluids

s4~dbeta(alpha.sens4,beta.sens4) ## Sensitivity PCR oral fluids

#########################################################

# covariance parameters with adjustment to positive range

#########################################################

covs13 <- u.covs13*uls13

covs24 <- u.covs24*uls24

covc13 <- u.covc13*ulc13

covc24 <- u.covc24*ulc24

# prior distribution of transformed covariances on (0,1) range

u.covs13 ~ dbeta(alpha.covs13,beta.covs13)

u.covs24 ~ dbeta(alpha.covs24,beta.covs24)

u.covc13 ~ dbeta(alpha.covc13,beta.covc13)

u.covc24 ~ dbeta(alpha.covc24,beta.covc24)

#upper limit

uls13 <- min(s1,s3) - s1*s3

ulc13 <- min(c1,c3) - c1*c3

uls24 <- min(s2,s4) - s2*s4

ulc24 <- min(c2,c4) - c2*c4

#correlation of test results between infected individuals (rhoD) and noninfected (rhoDc)

rhoD13 <- covs13 / sqrt(s1*(1-s1)*s3*(1-s3))

rhoD24 <- covs24 / sqrt(s2*(1-s2)*s4*(1-s4))

rhoDc13 <- covc13 / sqrt(c1*(1-c1)*c3*(1-c3))

rhoDc24 <- covc24 / sqrt(c2*(1-c2)*c4*(1-c4))

###################

#Parallel Se and Sp

###################

covsepar12 <- 0

covsepar14 <- 0

covsepar34 <- 0

covsepar23 <- 0

covsepar13 <- rhoD13*sqrt(s1*(1-s1)*(s3)*(1-s3))

covsepar24 <- rhoD24*sqrt(s2*(1-s2)*(s4)*(1-s4))

covsppar12 <- 0

covsppar14 <- 0

covsppar34 <- 0

covsppar23 <- 0

covsppar13 <- rhoDc13*sqrt(c1*(1-c1)*(c3)*(1-c3))

covsppar24 <- rhoDc24*sqrt(c2*(1-c2)*(c4)*(1-c4))

Separ <- 1-(1-s1)*(1-s2)*(1-s3)*(1-s4)-covsepar12*(1-s3)*(1-s4)-covsepar13*(1-s2)*(1-s4)-covsepar14*(1-s2)*(1-s3)-covsepar23*(1-s1)*(1-s4)-covsepar24*(1-s1)*(1-s3)-covsepar34*(1-s1)*(1-s2)

Separ13 <- 1-(1-s1)*(1-s3)-covsepar13

Separ24 <- 1-(1-s2)*(1-s4)-covsepar24

Separ12 <- 1-(1-s1)*(1-s2)

Separ34 <- 1-(1-s3)*(1-s4)

Sppar <- c1*c2*c3*c4+covsppar12*c3*c4+covsppar13*c2*c4+covsppar14*c2*c3+covsppar23*c1*c4+covsppar24*c1*c3+covsppar34*c1*c2

Sppar13 <- c1*c3+covsppar13

Sppar24 <- c2*c4+covsppar24

Sppar12 <- c1*c2

Sppar34 <- c3*c4

}
